# Endobody: Genetically Encodable Nanobody‐CPP Chimeras for Degradation of Membrane and Extracellular Proteins

**DOI:** 10.1002/advs.76075

**Published:** 2026-06-23

**Authors:** Chengjian Zhou, Huiping He, Simin Xia, Xi Chen

**Affiliations:** ^1^ Laboratory of Chemical Biology and Frontier Biotechnologies The HIT Center for Life Sciences Harbin Institute of Technology Harbin China; ^2^ Faculty of Life Sciences and Medicine Harbin Institute of Technology Harbin China

**Keywords:** cell‐penetrating peptide (CPP), epidermal growth factor receptor (EGFR), human epididymis protein 4 (HE4), nanobody, targeted protein degradation

## Abstract

Membrane protein degraders (MPDs) like LYTACs are emerging tools for targeting disease‐relevant membrane proteins, but their reliance on specific endocytic receptors limits their applicability across diverse cell types. Here, we present a structurally concise and genetically encodable class of degraders, nanobody‐cell‐penetrating peptide (CPP) chimeras, termed endobodies. We demonstrated that a genetically fused CPP is sufficient to mediate the internalization and subsequent degradation of membrane or extracellular proteins recognized by the nanobody. Using this platform, we achieved targeted degradation of membrane proteins including EGFR, PD‐L1, and HER2 in cancer cells. Importantly, the undruggable serum HE4, an ovarian cancer marker which could not be addressed by small‐molecule degraders, was also effectively depleted. Additionally, we first showcased the possibility to simultaneously degrade both extracellular and membrane proteins using a bispecific endobody. To further eliminate endosomal escapees, we engineered a panel of enhanced endobodies incorporating an additional proteasome‐targeting domain (PTD), resulting in more robust degradation. Notably, EGFR depletion by an enhanced endobody suppressed lung cancer cell proliferation and tumor growth in vivo. Collectively, endobody is an innovative class of MPDs, offering promising therapeutic potential.

## Graphical abstract

We introduced genetically encodable, receptor‐independent nanobody‐CPP chimeras, termed endobodies, as robust and modular membrane protein degraders. Additionally, proteasome‐targeting domain (PTD)‐tethered endobody demonstrates further enhanced degradation potency. Using this platform, we achieved targeted degradation of membrane proteins including EGFR, PD‐L1, and HER2, as well as the extracellular undruggable HE4 in serum. Notably, EGFR depletion suppressed lung cancer cell proliferation and tumor growth in vivo.

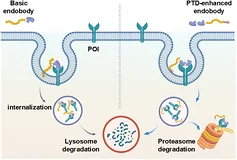

## 1 Introduction

Targeted protein degradation (TPD) is emerging as a promising modality in drug discovery with expanded druggable space [1, 2, 3, 4]. Mechanistically, TPD modalities can be broadly categorized into three major pathways: endosomal‐lysosomal system (ELS)‐based, autophagy‐based, and ubiquitin‐proteasome system (UPS)‐based degradation [5]. The first two are associated with lysosomal clearance [1], while all rely on protease‐mediated degradation. Among these approaches, membrane protein degradation has attracted great attention because of the clinical relevance of many membrane proteins as validated drug targets [6].

**FIGURE 1:**
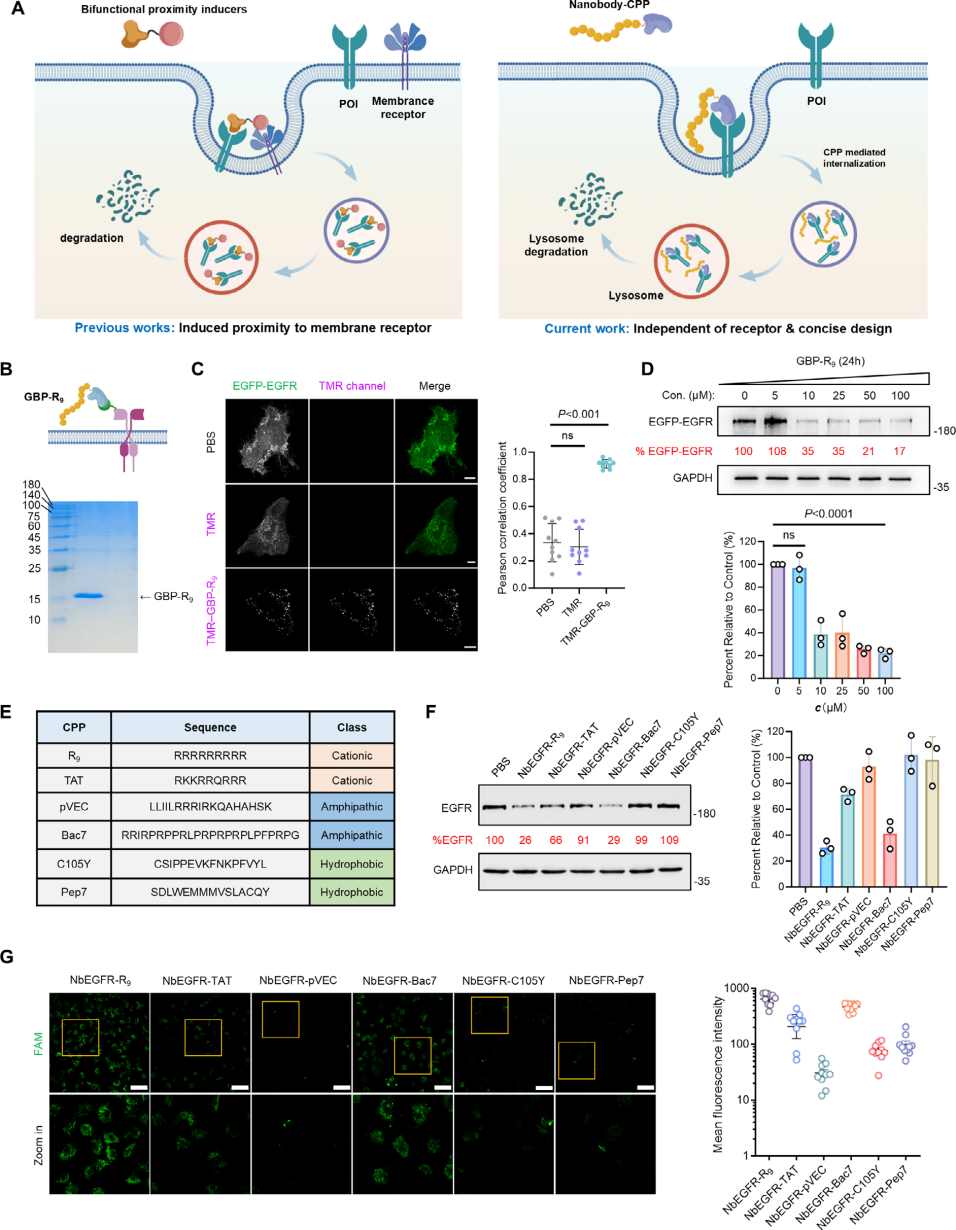
Conceptual schematic of endobody design and comparative evaluation of CPPs on degradation potency. (A) Schematic overview of the Nb‐CPP chimeras for degradation of cell membrane proteins as compared to conventional LYTACs. (B) Preparation and SDS‐PAGE characterization of a model endobody GBP‐R_9_ capable of degrading EGFP‐fused membrane protein. (C) Confocal micrographs reveal the colocalization between 5‐carboxytetramethylrhodamine (5‐TMR)‐labeled GBP‐R_9_ (10 µM, 24 h, 2×PBS washout) and EGFP‐EGFR and the formation of puncta inside live HeLa cells. Pearson's correlation coefficient (PCC) analysis was given (*n* = 10 cells). (D) Western blot analysis reveals dose‐dependent degradation of EGFP‐EGFR (*n* = 3 experiments). (E) List of three types of CPPs evaluated including cationic (R_9_, TAT), amphipathic (pVEC, Bac7), and hydrophobic (C105Y, Pep7) CPPs. (F) Western blot analysis of the potency of NbEGFR‐CPP chimeras for degradation of EGFR in HeLa cells (*n* = 3 experiments). (G) Left: Representative confocal fluorescence micrographs of HeLa cells treated with 5‐FAM‐labeled NbEGFR‐CPP chimeras (1 µM, 24 h, 2×PBS washout) revealing varied degrees of internalization (*n* = 10 cells). Scale bars: 50 µm. Right: Average fluorescence intensity (a.u.) for each group (*n* = 10 cells).

Lysosome‐targeting chimeras (LYTACs) [7] represent a pioneering class of membrane protein degraders (MPDs) [8]. They employ an antibody to bind with the target membrane protein and a chemically conjugated polysaccharide to engage an endocytic receptor, thereby directing the complex to lysosomal degradation via the ELS pathway. Building on the LYTAC concept, a range of MPD modalities have been developed to target both membrane and extracellular proteins, including KineTAC [9], AbTAC [10], PROTAB [11], GlueTAC [12], DENTACs [13], IFLD [14], and so on [15, 16, 17, 18]. While these platforms vary in molecular design, most rely on endocytic receptor engagement to facilitate endosomal uptake and subsequent lysosomal degradation (Figure 1A, left).

However, the aforementioned MPD modalities are constrained by their dependence on specific overexpressed endocytic receptors, limiting their applicability across diverse cell types. Moreover, constructs like LYTACs often involve structural complexity due to chemical conjugation with receptor ligands such as glycans. These design requirements pose challenges for scalability, genetic encoding, and immunogenicity. Therefore, there remains a strong need for alternative degradation platforms that are structurally streamlined, genetically encodable, and minimally immunogenic.

Cell‐penetrating peptides (CPPs) are short peptides (<30 amino acids) capable of crossing biological membranes to deliver bioactive cargos into cells [19], a process typically accompanied by inevitable lysosomal degradation [20, 21]. Inspired by this property, we hypothesized that genetically fusing a CPP with a nanobody, the variable domain of a heavy‐chain‐only antibody [22, 23], may directly drive endosomal entrapment and lysosomal clearance of nanobody‐bound membrane or extracellular proteins. This strategy will thus eliminate the need for additional endocytic receptor ligand [7] or lysosomal sorting signals [12]. We propose that such nanobody‐CPP chimeras, which we term endobodies, will represent a structurally concise, genetically encodable, and receptor‐independent class of MPDs (Figure 1A, right). In this study, we demonstrate their modular design, potential for further elaboration, and application in degrading disease‐associated membrane and extracellular proteins relevant to cancer therapy.

## 2 Results and Discussion

### 2.1 Comparative Evaluation of CPPs for Mediating Membrane Protein Degradation

To start, we designed a model endobody, GBP‐R_9_, which comprises a green fluorescent protein binding protein (GBP) nanobody (K_d_ 1.2 nM) [24] and a C‐terminal R_9_ cell‐penetrating peptide, thus can potentially degrade EGFP‐fused membrane proteins (Figure 1B). Confocal micrographs revealed that GBP‐R_9_ shows a strong colocalization with EGFP‐fused epidermal growth factor receptor (EGFR) along with the formation of small puncta inside living cells, suggesting that GBP‐R_9_ could bring EGFP‐EGFR into live cells via endocytosis (Figure 1C). Western blot (WB) analysis confirmed that GBP‐R_9_ is capable of degrading EGFP‐EGFR in a dose‐dependent fashion, suggesting that our conceptual design of Nb‐CPP chimera as a structurally minimal yet effective degrader (Figure 1D).

Next, we were motivated to evaluate different types of CPPs regarding their potency on membrane protein degradation. Since we were primarily interested in the degradation of EGFR which is often overexpressed in many cancer cell surfaces promoting uncontrolled cell division [25], we designed and prepared a panel of NbEGFR‐CPP chimeras comprising an EGFR‐targeting nanobody [26] (NbEGFR). These NbEGFR‐CPP chimeras were prepared from NbEGFR‐CPP‐intein‐His_6_ precursor via expressed protein ligation (EPL) by cleavage using L‐cysteine in the presence of sodium 2‐mercaptoethanesulfonate (MENSNa) and 4‐mercaptophenylacetic acid (MPAA) (Figure S1). We evaluated three types of CPPs including cationic (R_9_, TAT), amphipathic (pVEC, Bac7), and hydrophobic (C105Y, Pep7) versions (Figure 1E). WB analysis reveals that aside from the two hydrophobic CPPs (C105Y & Pep7) which show no detectable degradation, three CPPs, that is, R_9_, TAT, and Bac7 belonging to either cationic or amphipathic CPPs triggers endogenous EGFR degradation, with cationic R_9_ demonstrating the highest potency followed by amphipathic Bac7 (Figure 1F).

To evaluate the internalization efficiency, we treated live HeLa cells with 5‐carboxyfluorescein (5‐FAM)‐labeled NbEGFR‐CPP chimeras, which revealed that R_9_ showed the highest fluorescence intensity inside live cells, followed by Bac7, consistent with their degradation potency (Figure 1G). We also analyzed 5‐FAM‐labeled NbEGFR‐CPP chimeras regarding their colocalization with early endosomes (mCherry‐Rab5a), which also showed that NbEGFR‐R_9_ demonstrated the highest colocalization (Figure S2A,B) suggesting the strongest endosomal entrapment. Notably, either R_9_ or GBP‐R_9_ exhibits negligible or insignificant cytotoxicity at concentrations up to 25 µM (Figure S2C,D), supporting the safe application of R_9_ sequence in endobody construction. Hence, we have identified R_9_ as an optimal CPP for designing of a Nb‐CPP chimera for degradation of membrane proteins.

### 2.2 Modular Endobody Enables Degradation of EGFR, PD‐L1, and HER2

**FIGURE 2:**
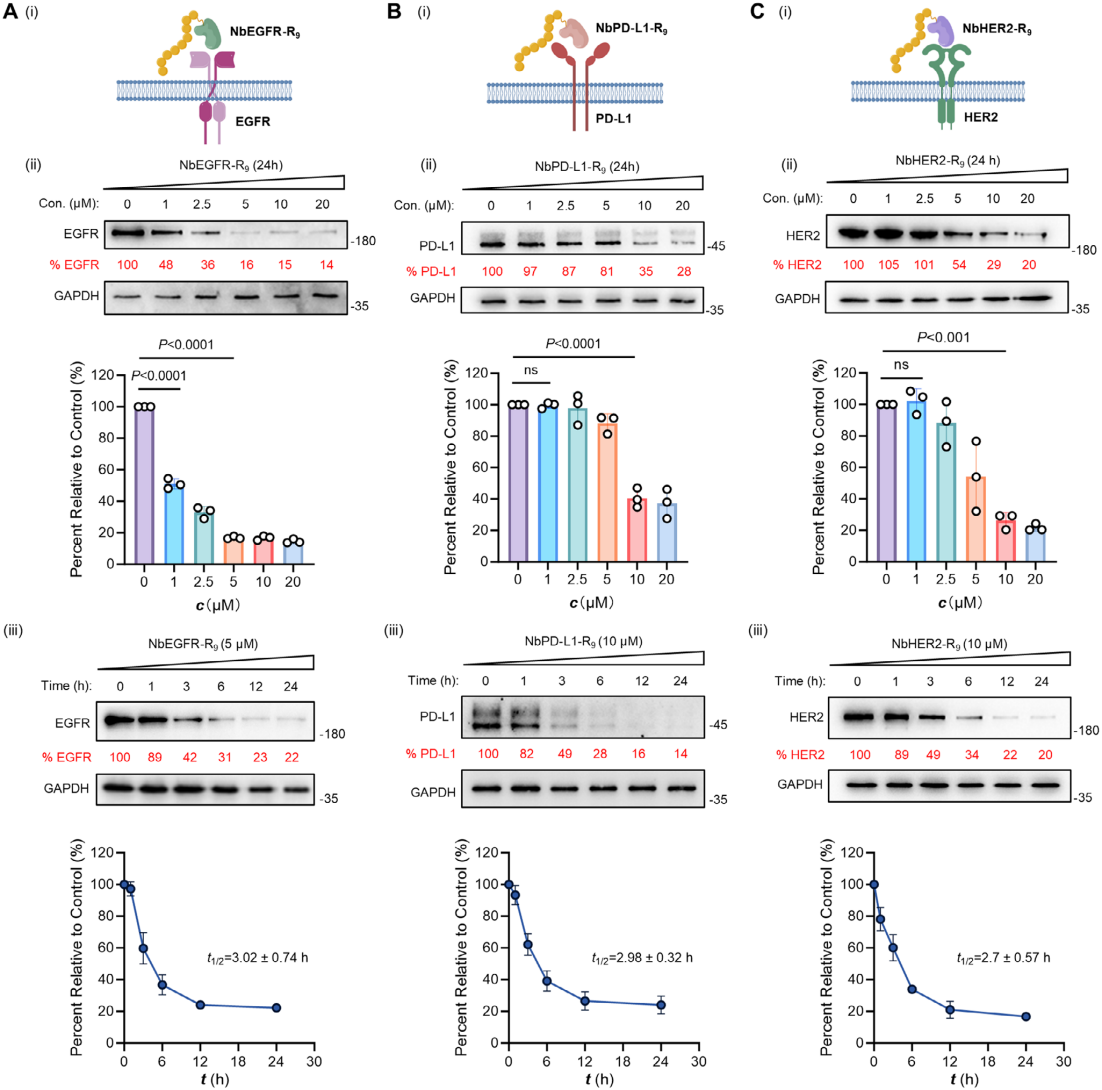
Modularity of endobodies: Degradation of different membrane proteins via exchange of the nanobody module. (A‐C) Degradation of EGFR by NbEGFR‐R_9_ in HeLa cells (A), degradation of PD‐L1 by NbPD‐L1‐R_9_ in MDA‐MB‐231 cells (B), and degradation of HER2 by NbHER2‐R_9_ in SK‐BR‐3 cells (C). For (i) in panels (A–C): Schematics of NbEGFR‐R_9_, NbPD‐L1‐R_9_, and NbHER2‐R_9_ endobodies. For (ii) in panels (A–C): Representative WB analysis and corresponding quantifications of NbEGFR/NbPD‐L1/NbHER2‐R_9_ mediated degradation of EGFR, PD‐L1, and HER2, respectively, after treatment of gradient concentrations of the drug for 24 h (*n* = 3 experiments). For (iii) in A‐C: WB analysis showing time‐dependent degradation of membrane proteins after treatment of given concentrations of degrader (*n* = 3 experiments). One‐sided independent Student's *t*‐test were used in all statistical analysis; ns: non‐significant.

We next assessed the general applicability and modularity of Nb‐CPP chimeras for degrading membrane proteins. First, we investigated the degradation profile of NbEGFR‐R_9_ for degradation of endogenous EGFR in live HeLa cells (Figure 2A_i). WB analysis revealed dose‐dependent degradation of EGFR with around 5 µM to achieve the maximal degree of degradation (Figure 2A_ii). Time‐course experiment revealed higher degree of degradation along with increased incubation time using 5 µM of NbEGFR‐R_9_ (Figure 2A_iii). We further confirmed that this degradation is due to the presence of CPP because a control construct, NbEGFR free of R_9_ could not be internalized nor induce EGFR degradation (Figure S3A,B).

Meanwhile, to evaluate whether the fused R_9_ moiety affects the antigen‑binding capacity of the nanobody, we performed immunoprecipitation (IP) assays using NbEGFR and NbEGFR‑R_9_ with HeLa cell lysates. IP results showed that both constructs pulled down comparable levels of EGFR, indicating that R_9_ fusion does not alter target binding (Figure S3C). In addition, to examine whether the cell‑penetrating peptide (CPP) itself influences membrane protein degradation, we conducted a free R_9_ competition assay by treating cells with NbEGFR‑R_9_ in the presence or absence of excess free R_9_ peptide. No significant differences in EGFR degradation were observed, potentially suggesting that the R_9_ moiety does not affect degradation through aggregation or non‑specific competition (Figure S3D).

Programmed death ligand 1 (PD‐L1) is an immune checkpoint protein frequently overexpressed on cancer cell surfaces to enable immune escape [27]. Thus, degradation of PD‐L1 in cancer cells could restore the antitumor immunity of T‐cells for killing cancer. Hence, we designed NbPD‐L1‐R_9_ endobody comprising a PD‐L1 nanobody [28] (NbPD‐L1) fused with a C‐terminal R_9_ CPP, and applied it with PD‐L1‐positive MDA‐MB‐231 cells (Figure 2B_i; Figure S4A). Similarly, WB analysis revealed dose‐dependent (Figure 2B_ii) and time‐dependent (Figure 2B_iii) degradation of PD‐L1. Again, NbPD‐L1 free of R_9_ could not induce PD‐L1 degradation (Figure S4B,C).

Human epidermal growth factor receptor 2 (HER2) is a tyrosine kinase receptor that is frequently overexpressed in breast cancer cells, driving tumor proliferation [29]. Targeted degradation of HER2 thus represents a promising therapeutic strategy. Therefore, we designed NbHER2‐R_9_ endobody comprising a nanobody against HER2 (NbHER2) [30] and a R_9_ peptide, and applied it to treat HER2‐positive SK‐BR‐3 breast cancer cells (Figure 2C_i; Figure S5A). NbHER2‐R_9_ induced dose‐dependent (Figure 2C_ii) and time‐dependent (Figure 2C_iii) degradation of the membrane protein HER2 with the maximal degradation degree observed at 24 h. In contrast, NbHER2 free of R_9_ failed to internalize and did not induce HER2 degradation (Figure S5B,C). In addition, NbEGFR‐R_9_ endobody, rather than control GBP‐R_9_ endobody, induces EGFR degradation in EGFR/PD‐L1 double‐positive MDA‐MB‐231 cells, while neither construct alters the level of another membrane protein PD‐L1, verifying that endobodies specifically degrade their recognized membrane protein targets without affecting unrelated ones (Figure S5D,E). These results underscore the modularity and potency of the endobody platform as a broadly applicable strategy for membrane protein degradation.

### 2.3 Mechanistic Investigation Reveals Clathrin‐dependent ELS Pathway

**FIGURE 3:**
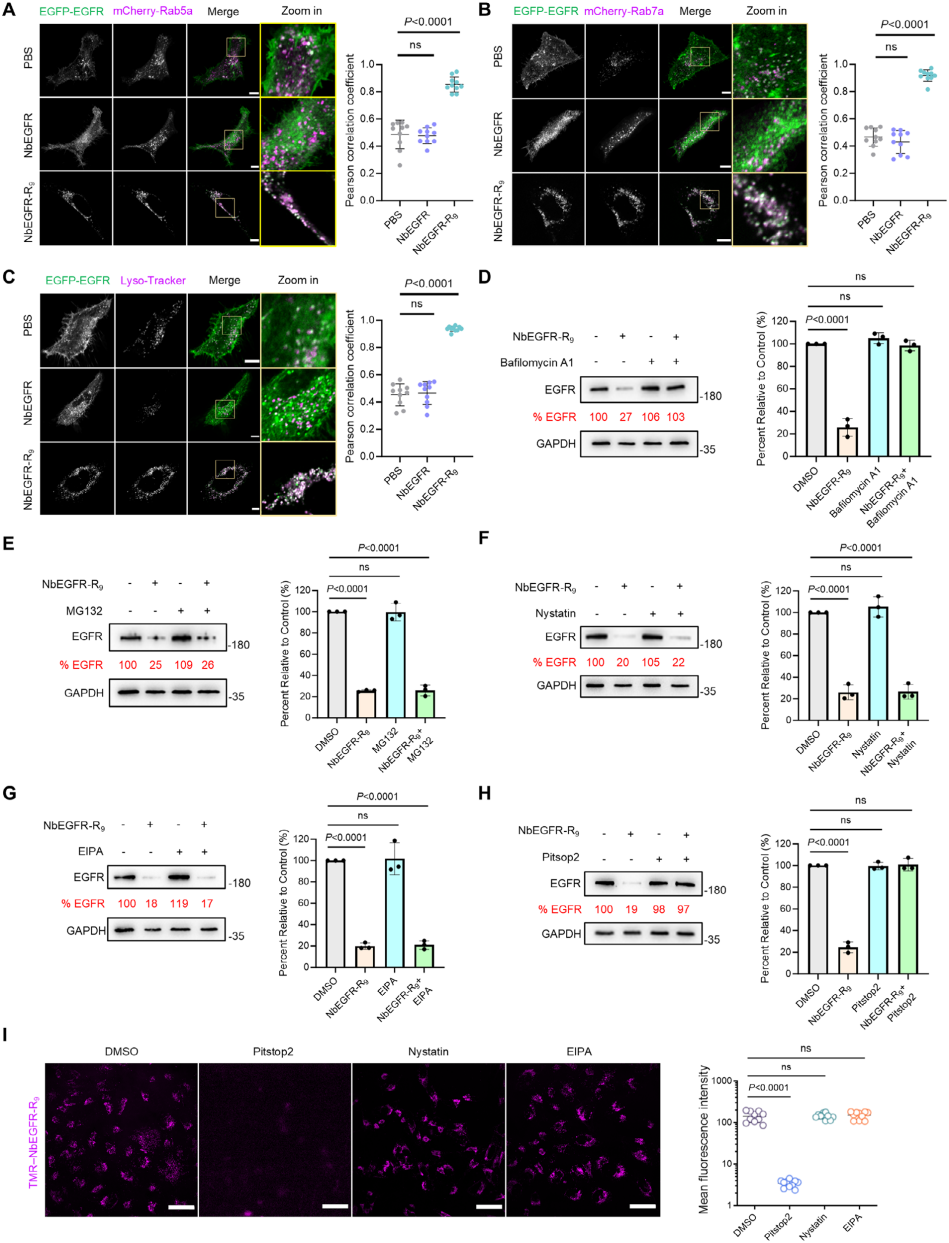
Mechanistic investigation: Clathrin‐dependent, caveolin‐independent ELS pathway. (A) Left: Confocal micrographs of HeLa cells co‐expressing EGFP‐EGFR (green) and mCherry‐Rab5a (early endosome marker, magenta) treated with NbEGFR‐R_9_, NbEGFR (Ctrl) or PBS (blank) revealed entrapment of EGFP‐EGFR in early endosomes only in the presence of NbEGFR‐R_9_. Right: PCC colocalization analysis between channels for each group (*n* = 10 cells). (B) Left: Confocal micrographs of HeLa cells co‐expressing EGFP‐EGFR (green) and mCherry‐Rab7a (late endosome marker, magenta) treated with NbEGFR‐R_9_, NbEGFR (Ctrl) or PBS (blank) revealed entrapment of EGFP‐EGFR in late endosomes only in the presence of NbEGFR‐R_9_. Right: PCC colocalization analysis between channels for each group (*n* = 10 cells). (C) Left: Confocal micrographs of Lyso‐Tracker (magenta) labeled HeLa cells expressing EGFP‐EGFR (green) treated with NbEGFR‐R_9_, NbEGFR (Ctrl) or PBS (blank) revealed entrapment of EGFP‐EGFR in lysosomes only in the presence of NbEGFR‐R_9_. Right: PCC colocalization analysis between channels for each group (*n* = 10 cells). (D) WB analysis revealed inhibited degradation in the presence of the lysosome inhibitor Bafilomycin A1 (100 nM). (E) WB analysis revealed no inhibition of degradation in the presence of proteasomal inhibitor MG132 (1 µM). (F) WB analysis revealed no inhibition of degradation in the presence of caveolae‐dependent endocytosis inhibitor Nystatin (20 µM). (G) WB analysis revealed no inhibition of degradation in the presence of macropinocytosis inhibitor EIPA (30 µM). (H) WB analysis revealed inhibition of degradation in the presence of clathrin‐mediated endocytosis inhibitor pitstop 2 (30 µM). (I) Left: Confocal micrographs of live HeLa cells incubated with Nystatin (20 µM), EIPA (30 µM), and pitstop 2 (30 µM) for 6 h before further adding 5‐TMR‐labled NbEGFR‐R_9_ (5 µM, 24 h, 2×PBS washes) revealed that only pitstop 2 prohibited internalization. Right: Quantification of average fluorescence intensities (a.u.) of each group (*n* = 10 cells). Scale bars: 50 µm; one‐sided independent Student's *t*‐test was used; ns: nonsignificant.

To elucidate the working mechanism of Nb‐CPP degraders, we first visualized the colocalization with endosomal‐lysosomal system (ELS) related organelles after internalization. Confocal micrographs revealed that the presence of NbEGFR‐R_9_ (5 µM, 24 h, 2×PBS washes) induced the internalization of EGFP‐EGFR and its colocalization with early endosomes (Figure 3A, mCherry‐Rab5a maker), late endosomes (Figure 3B, mCherry‐Rab7a marker), and lysosomes (Figure 3C, Lyso‐Tracker). Further, we found that NbEGFR‐R_9_ induced degradation was fully inhibited in the presence of the lysosome inhibitor, Bafilomycin A1 (100 nM) but not the proteasome inhibitor MG132 (1 µM, Figures 3D,E). The evidences confirmed endosomal‐lysosomal system (ELS)‐based but not UPS‐based protein degradation pathway.

Previous studies have indicated that certain specific signaling pathways, rather than individual proteins, mediate the activity of ELS‐based MPDs [31]. Thus, instead of genome‐wide screening, we directly inhibited well‐characterized endolysosomal sorting pathways to further elucidate the action mechanism of endobodies. CPP‐based internalization can proceed via caveolae‐dependent endocytosis, clathrin‐mediated endocytosis, or macropinocytosis [21]. In this regard, we pre‐treated live HeLa cells with caveolae‐dependent endocytosis inhibitor Nystatin (20 µM), clathrin‐mediated endocytosis inhibitor pitstop 2 (30 µM), or macropinocytosis inhibitor 5‐(*N*‐ethyl‐*N*‐isopropyl)amiloride (EIPA, 30 µM). WB analysis revealed that only pitstop 2 but Nystatin nor EIPA inhibited the degradation of EGFR by NbEGFR‐R_9_ endobody (Figures 3F–H). Furthermore, confocal microscopy demonstrated that pitstop 2 but Nystatin nor EIPA inhibited the internalization of 5‐TMR‐labeled NbEGFR‐R_9_ (Figure 3I). Hence, these studies verified clathrin‐dependent, caveolin‐independent ELS pathway for Nb‐R_9_ endobody induced protein degradation.

### 2.4 Targeted Degradation of the Extracellular Undruggable HE4 by a HE4‐specific Endobody

**FIGURE 4:**
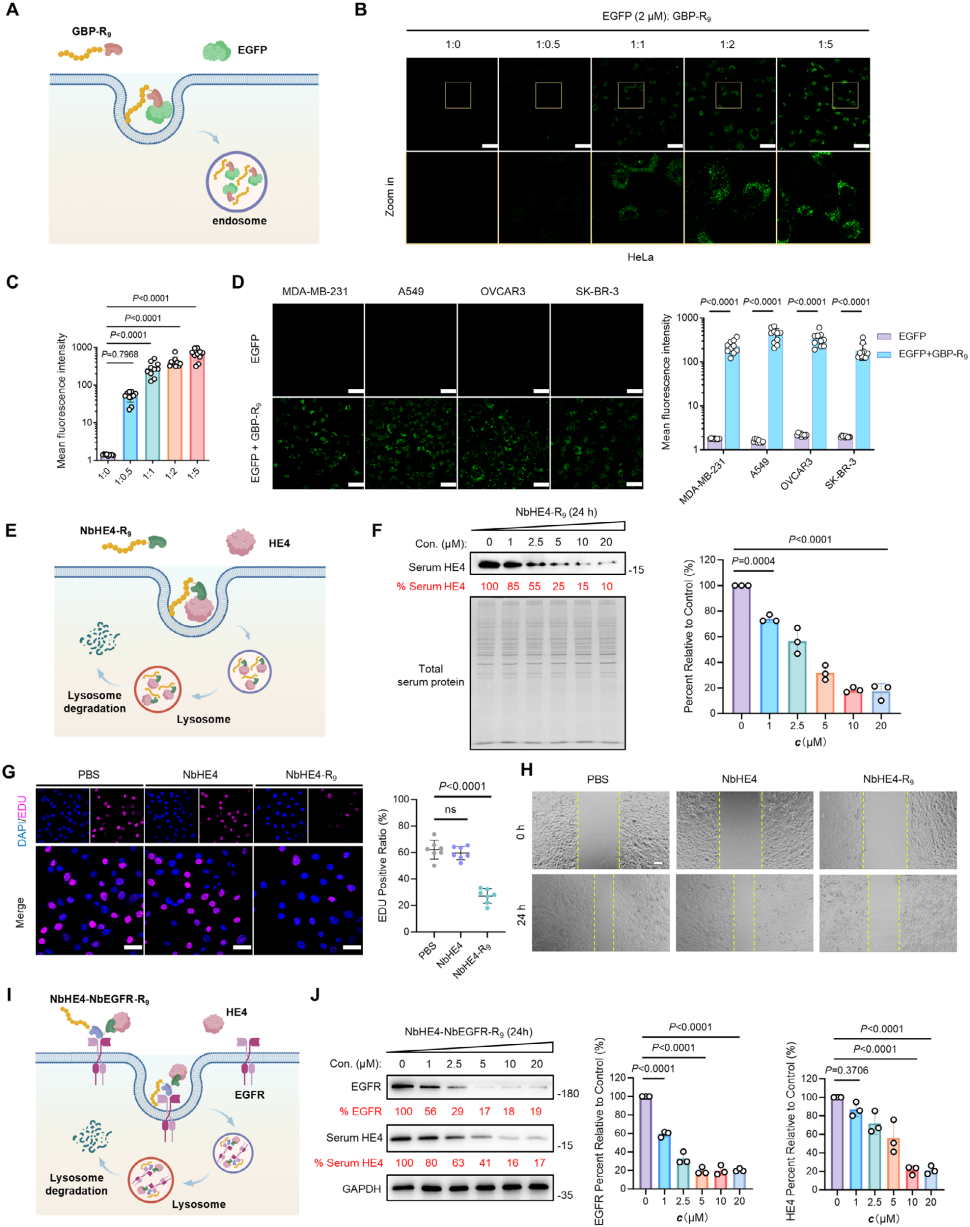
Endobodies enable degradation of extracellular proteins. (A) Schematic of serum EGFP internalization mediated by GBP‐R_9_ model endobody. (B‐C) Representative confocal micrographs of HeLa cells in medium containing EGFP (2 µM) plus increasing equivalents of GBP‐R_9_ endobody (0/0.5/1/2/5 equiv.); scale bars: 50 µm. Statistical analysis: *n* = 10 cells, mean ± SD. (D) Confocal micrographs revealed efficient internalization of EGFP (2 µM) in the presence of GBP‐R_9_ (5 equiv.) in different cell lines; scale bars: 50 µm. Statistical analysis: *n* = 10 cells, mean ± SD. (E) Schematic of serum HE4 internalization and lysosomal degradation mediated by NbHE4‐R_9_ endobody. (F) WB analysis revealed dose‐dependent degradation of serum HE4 in the presence of NbHE4‐R_9_ endobody (note that total serum protein was used as internal control). Statistical analysis: *n* = 3 experiments; mean ± SD; Student's t‐test used. (G) EdU assay revealed inhibited proliferation of ovarian cancer cell OVCAR3 by NbHE4‐R_9_ (10 µM) but not NbHE4 (Ctrl, 10 µM) nor PBS (blank); scale bars: 10 µm. Statistical analysis: *n* = 7 fields; mean ± SD; Student's t‐test used. (H) Scratch‐wounding assay revealed inhibited OVCAR3 cell migration in the presence of NbHE4‐R_9_ (10 µM) but not NbHE4 (Ctrl, 10 µM) nor PBS (blank); scale bars: 100 µm. (I) Schematic of a bispecific Nb‐CPP chimera for simultaneous degradation of both extracellular and membrane proteins. (J) The bispecific endobody, NbHE4‐NbEGFR‐R_9_ enables simultaneous degradation of both extracellular HE4 and membrane EGFR in live OVCAR3 cells according to WB analysis. Statistical analysis: *n* = 3 experiments; mean ± SD; Student's *t*‐test used.

Motivated by the above results, we envisioned that endobody may also mediate the internalization and degradation of non‐membrane anchored serum or extracellular proteins, such as those considered undruggable thus challenging for small‐molecule ligand‐based degraders (Figure 4A). To test this hypothesis, we added gradient equivalents (0/0.5/1/2/5 equiv.) of GBP‐R_9_ endobody in HeLa cell culture medium containing EGFP (2 µM). Confocal micrographs revealed increased internalization of EGFP along with the enhanced equivalents of GBP‐R_9_ (Figures 4B‐C). To test the general applicability of this delivery, we also evaluated other cell lines including MDA‐MB‐231, A549, OVCAR3, and SK‐BR‐3 cells. Similarly, GBP‐R_9_ (10 µM) mediated efficient internalization of serum EGFP (2 µM) into these different cell lines (Figure 4D). Hence, endobody is able to mediate serum protein internalization, suggesting the possibility for degradation of soluble extracellular proteins by endobodies.

Human epididymis protein 4 (HE4) is a recently recognized cancer marker overexpressed in some particular cancer types such as ovarian, pancreatic, breast, and uterine cervical cancers [32], and the degradation of it was suggested to demonstrate therapeutic potential [33, 34]. Although HE4 is also present intracellularly, a large portion of HE4 is secreted into the serum, where it reactivates cell proliferation through multiple pathways.

Unfortunately, HE4 is free of a ligand‐binding pocket rendering it inaccessible by small molecule‐based degraders. Therefore, we treated ovarian OVCAR3 cell culture with NbHE4‐R_9_ endobody comprising a nanobody targeting HE4 [35] (NbHE4) and a R_9_ peptide (Figure S6) in order to deplete serum HE4 (Figure 4E). WB analysis revealed dose‐dependent degradation of HE4 with maximal around 90 % of depletion (Figure 4F). Importantly, degradation of serum HE4 significantly inhibited OVCAR3 cell proliferation and migration according to 5‐ethynyl‐2'‐deoxyuridine (EdU) proliferation assay (Figure 4G) and scratch‐wounding migration assay (Figure 4H), respectively. Therefore, endobody is also capable to deplete disease‐driving extracellular proteins, holding potential for designing innovative nanobody‐based therapeutics.

### 2.5 Simultaneous Extracellular and Membrane Protein Degradation Using a Bispecific Endobody

As endobodies are genetically encodable, we envisioned the possibility to design a bispecific endobody encompassing a bispecific nanobody module for simultaneous targeted degradation of both a serum protein and an extracellular protein. In this regard, we designed and prepared NbHE4‐NbEGFR‐R_9_ bispecific endobody (Figure S7), with the aim for co‐depletion of both serum HE4 and membrane EGFR (Figure 4I). Delightfully, WB analysis confirmed dose‐dependent depletion of both serum HE4 and EGFR in live OVCAR3 cell (Figure 4J). To the best of our knowledge, this represents the first example of simultaneous degradation of both a serum protein and a membrane protein using a single degrader. Thus, this strategy is expected to hold potential for design of innovative degraders showing synergistic pharmacological effect.

### 2.6 Design of PTD‐Enhanced Endobodies Demonstrating Higher Degradation Potency

**FIGURE 5:**
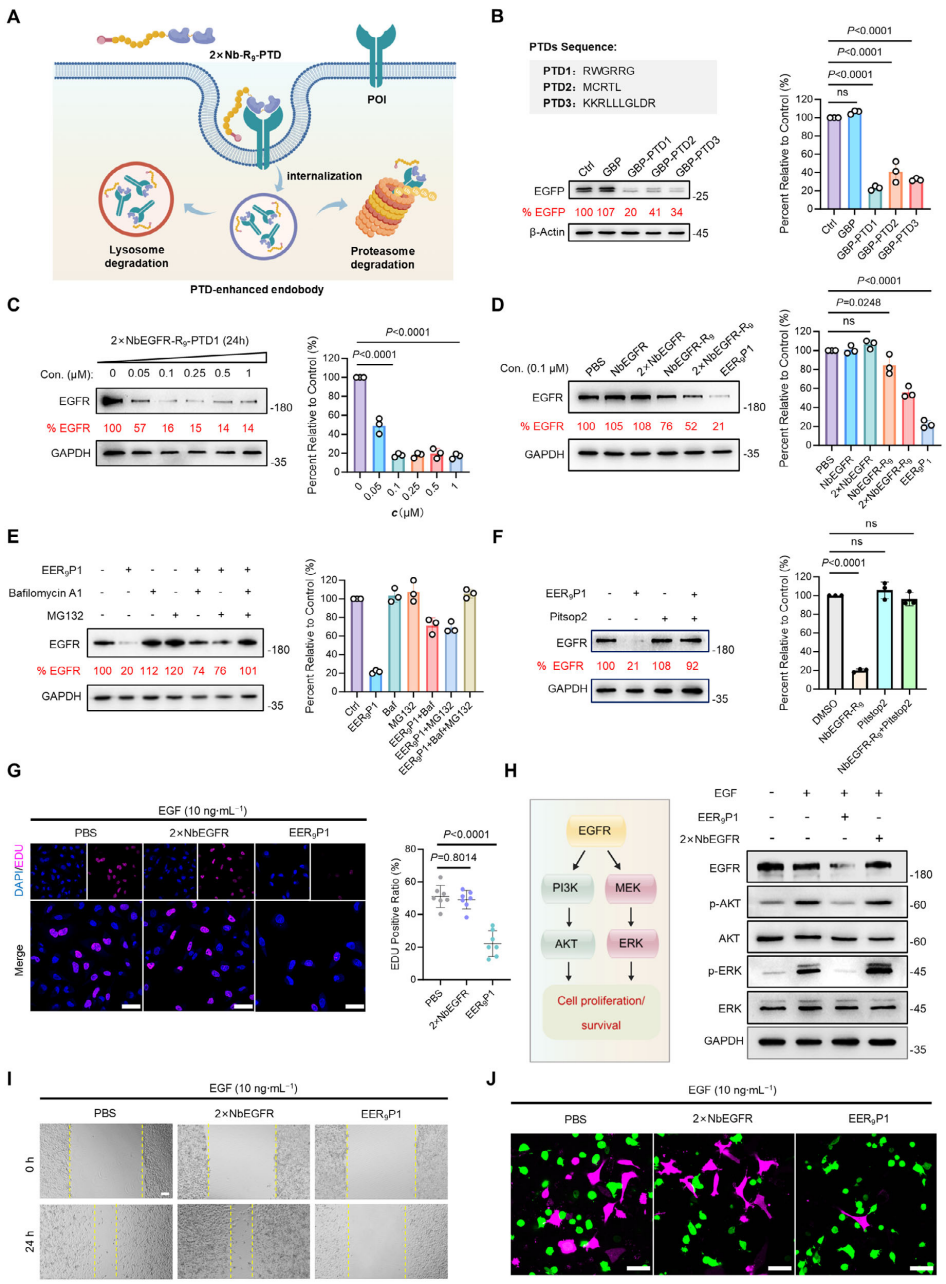
PTD‐enhanced endobody allows more potent degradation of EGFR. (A) Schematic of proteasome‐targeting domain (PTD)‐tethered endobody with enhanced degradation potency. (B) WB analysis revealed different efficiency of three PTD sequence for mediating intracellular EGFP degradation by GBP‐mCherry‐PTD1/2/3; Ctrl: EGFP alone. (C) WB analysis revealed enhanced potency for PTD1‐tethered NbEGFR‐R_9_ endobody (i.e., 2×NbEGFR‐R_9_‐PTD1, or EER_9_P1) to induce EGFR degradation at low‐nanobody concentration. (D) Comparison of EER_9_P1 with NbEGFR‐R_9_ endobody and their respective non‐functional controls (2×NbEGFR and NbEGFR) for the degradation of EGFR via WB analysis. (E) WB analysis revealed that Bafilomycin A1 (100 nM) or MG132 (1 µM) only partially inhibited EGFR degradation by EER_9_P1 (100 nM) while Bafilomycin A1 and MG132 together fully inhibited EGFR degradation, confirming the involvement of both ELS and UPS pathways. (F) WB analysis revealed the inhibition of degradation by pistop2 (30 µM), suggesting clathrin‐mediated lysosomal degradation. (G) EdU assay revealed inhibition of HeLa cell proliferation in the presence of EER_9_P1 but not 2×NbEGFR (Ctrl) nor PBS (blank, *n* = 7 fields per group). (H) WB analysis of the PI3K/AKT pathway (phosphorylated AKT, p‐AKT) and MAPK/ERK pathway (phosphorylated ERK, p‐ERK) after EGFR degradation by EER_9_P1 (100 nM) revealing inhibition of the phosphorylation of both AKT and ERK. For all statistical analysis of WB: *n* = 3 experiments; mean ± SD; Student's t‐test used; ns: non‐significant. (I) Scratch‐wounding assay revealed inhibited HeLa cell migration in the presence of EER_9_P1 but not 2×NbEGFR (Ctrl) at 100 nM concentration; scale bars: 100 µm. (J) EER_9_P1 selectively kills EGFR‐positive HeLa cells (magenta, marked by mCherry) over EGFR‐negative HEK293T cells (green, marked by EGFP) according to live cell fluorescence microscopy; scale bars: 100 µm.

Internalized membrane proteins may potentially undergo partial endosomal escape to cytosol as observed in our previous experiments (Figure S2), thus reducing lysosomal degradation and comprising degradation potency. Therefore, we envisioned that tethering of an additional proteasome‐targeting domain (PTD) would further deplete the escaped fraction, thus enhancing the degradation potency (Figure 5A). Hence, we first evaluated three PTD sequences [36], namely PTD1 (RWGRRG), PTD2 (MCRTL), and PTD3 (KKRLLLGLDR), which are fused with GBP to create three GBP‐mCherry‐PTD1/2/3 degraders. Cotransfection of EGFP and GBP‐mCherry‐PTD1/2/3 and WB analysis of EGFP degradation revealed that GBP‐mCherry‐PTD1 exhibited the highest degradation efficiency (Figure 5B).

Therefore, we designed a PDT1‐enhanced endobody, 2×NbEGFR‐R_9_‐PTD1 (EER_9_P1), which comprises an additional C‐terminal PTD1 sequence for proteasomal targeting as well as a bivalent NbEGFR nanobody for increased binding avidity which also enhances degradation (Figure S8). We were delighted to observe the boosted degradation potency of EER_9_P1 which induced EGFR degradation at low nanobody concentration (Figure 5C). We showed that EER_9_P1 triggers much higher degradation efficacy compared to NbEGFR‐R_9_ endobody as well as 2×NbEGFR‐R_9_ bivalent endobody at 100 nM concentration (Figure 5D). Mechanistic study using the proteasome inhibitor MG132 and the lysosome inhibitor Bafilomycin A1 reveals the participation of both ELS and UPS degradation pathways (Figure 5E). Further study using different endocytosis inhibitors confirmed clathrin‐mediated ELS pathway because pitstop2 (Figure 5F) but not Nystatin or EIPA fully inhibited EGFR degradation (Figure S9).

Motivated by above results, we examined cellular responses and downstream signaling pathways related to cell proliferation and survival upon EGFR degradation by EER_9_P1 in HeLa cells. Therefore, we performed EdU assay which confirms inhibited cell proliferation (Figure 5G). Additionally, WB analysis of EGFR‐related pathways further revealed inhibited phosphorylation of AKT and ERK (Figure 5H). Moreover, scratch‐wounding assay revealed the inhibition of cell migration after treatment with EER_9_P1 (100 nM) for 24 h, but not 2×NbEGFR nor PBS (Figure 5I). We demonstrated that this proliferative inhibition occurs selectively in EGFR‐positive rather than in EGFR‐negative cells (Figure 5J). Hence, PTD1‐enhanced endobody could significantly enhance the degradation potency by hijacking endosomal escape, and also highlighting the therapeutic value of PTD1‐enhanced endobody, such as EER_9_P1.

To demonstrate the general applicability of this PTD1‐tethered enhanced endobody strategy, we further designed and prepared three additional enhanced endobodies, including 2×NbPD‐L1‐R_9_‐PTD1, 2×NbHER2‐R_9_‐PTD1, 2×NbHE4‐R_9_‐PTD1 (Figure S10). Delightfully, all these constructs demonstrate increased degradation potency compared to their respective standard endobody version (Figure S11), highlighting the modularity and general applicability of this enhanced endobody strategy.

### 2.7 A PTD‐Enhanced Endobody Suppresses EGFR‐Positive Tumor Growth In Vivo

**FIGURE 6:**
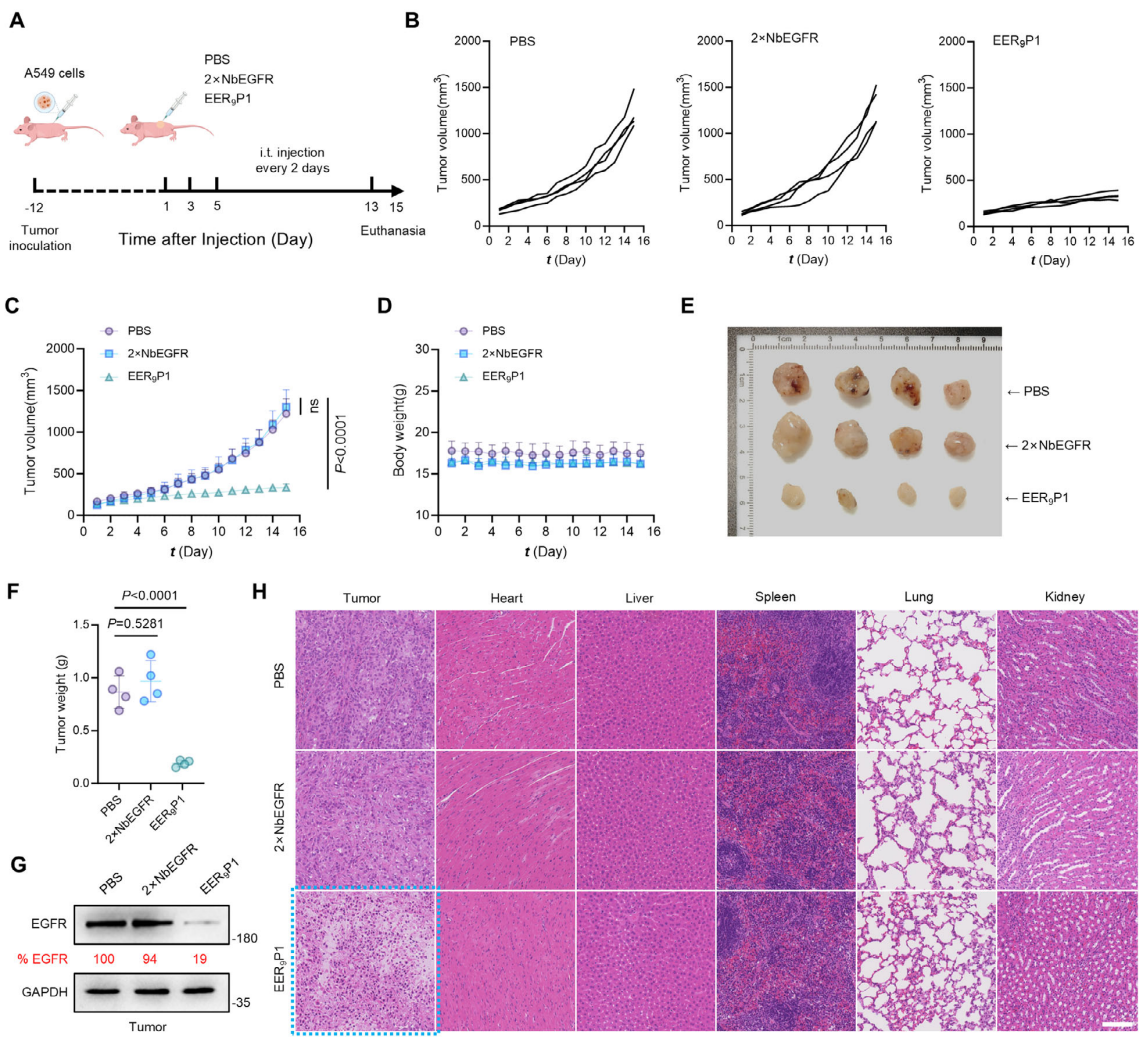
PTD‐enhanced endobody EER_9_P1 suppresses growth of lung cancer xenograft tumor in vivo. (A) Schematic overview of xenograft lung cancer mice model generation and drug administration in BALB/c nude mice. ∼6×10^6^ A549 cells in 0.1 mL of PBS/Matrigel solution were subcutaneously injected into the axillary region of the BALB/c nude mice to allow tumor generation. Drugs were intratumorally injected every two days. (B) Tumor growth curve of each individual mouse for PBS (blank), 2×NbEGFR (Ctrl, 10 mg∙kg^−1^), and EER_9_P1 (10 mg∙kg^−1^) injected groups (*n* = 4 mice per group). (C) Statistical quantification of the tumor growth for different groups. Statistical analysis: mean ± SD; Student's *t*‐test used; ns: non‐significant. (D) Body weight curves for each group of mice. Mean ± SD is shown. (E) Photographs of the dissected tumor of each mouse 15 days after the first drug injection. (F) Statistical analysis of the tumor weight of each mice group: Mean ± SD; Student's *t*‐test used; ns: non‐significant. (G) WB analysis of the dissected tumor tissue reveals degradation of EGFR in tumor tissue. (H) H&E staining of tumor sections revealed apoptosis induction in the EER_9_P1‐treated group, with no detectable apoptotic features observed in other treatment groups or in major healthy organs.

Therefore, we next evaluated the antitumor activity of EER_9_P1 in vivo using A549 lung cancer xenograft mice model (Figure 6A). After the tumor size reached around 6‐7 mm in diameter and started to grow steadily, EER_9_P1 (10 mg∙ml^−1^), 2×NbEGFR (Ctrl, 10 mg∙ml^−1^), or PBS (blank) was intratumorally injected every two days. Individual tumor volume growth curves show that both PBS‐injected blank group and 2×NbEGFR‐injected control group all show similar steady tumor growth, suggesting the bivalent 2×NbEGFR nanobody alone has no effect on tumor suppression (Figure 6B, left and middle). In contrast, mice injected with EER_9_P1 endobody demonstrates significant suppression of tumor growth (Figure 6B, right). Statistical quantification of tumor growth curves revealed that the tumor sizes of 2×NbEGFR‐injected group show no significant differences compared to the blank group 15 days post‐injection whereas the tumor sizes of EER_9_P1‐injected group were all significantly smaller (Figure 6C).

Meanwhile, recorded mice body weight shows almost no change over 15 days for all groups indicating that both EER_9_P1 and 2×NbEGFR are non‐toxic in vivo, which is reasonable because they in principle only target EGFR‐positive tumors but not EGFR‐negative healthy tissues (Figure 6D). Dissected tumors further revealed the much smaller sizes for EER_9_P1‐injected group but not control or blank groups (Figures 6E‐F). WB analysis of the digested tumor tissues confirmed degradation of EGFR in tumor tissues (Figure 6G). Hematoxylin‐eosin (H&E) staining confirmed apoptosis induction in the tumor tissue of EER_9_P1‐treated group but not in the blank and control group nor in the healthy tissues including heart, liver, kidney, lung, and spleen (Figure 6H).

To further verify the low systemic toxicity of EER9P1 in vivo, we evaluated its antitumor efficacy in A549 lung cancer xenograft mice via intravenous injection (Figure S12A). After the tumor size reached around 6‐7 mm in diameter and started to grow steadily, EER_9_P1 (3 mg∙ml^−1^), EER_9_P1 (5 mg∙ml^−1^), or PBS (blank) was intravenously injected every two days. Individual tumor volume growth curves show that PBS‐injected blank group show steady tumor growth while EER_9_P1‐injected groups demonstrate significant suppression of tumor growth (Figure S12B). Statistical quantification revealed that the tumor sizes of EER_9_P1‐injected groups were all significantly smaller (Figure S12C). Meanwhile, recorded mice body weight show almost no change over 15 days for all groups indicating that EER_9_P1 is non‐toxic in vivo with low systemic toxicity (Figure S12D). Dissected tumors showed markedly smaller sizes in the EER_9_P1‐injected groups but not the blank group (Figure S12E,F). Finally, we determined the serum stability of EER_9_P1, indicating a half‐life of approximately 12 h, consistent with that of typical nanobody chimeras (Figure S12G). Collectively, the PTD‐enhanced endobody, EER_9_P1, shows promising selective antitumor activity in vivo with minimal cytotoxicity to healthy tissues.

## 3 Conclusion

In summary, we developed genetically encodable nanobody‐CPP chimeras, designated endobodies, as an innovative class of membrane and extracellular protein degraders. Endobodies are succinct in composition, comprising solely a nanobody module and a CPP peptide, without requiring additional endocytic receptor ligand. Among the CPPs evaluated, the linear R_9_ peptide conferred the highest degradation potency. Mechanistic investigations revealed that endobody‐mediated degradation proceeds via a clathrin‐dependent, caveolin‐ and macropinocytosis‐independent ELS degradation pathway. To address the endosomal escaped fractions of membrane proteins, we also designed PTD‐enhanced endobodies bearing an additional proteasomal targeting sequence, resulting in further improved degradation performance.

We showcased the modularity of this degradation strategy by exchanging the nanobody module to target alternative membrane proteins, including EGFR, PD‐L1, and HER2. Leveraging the capability of CPP to delivery extracellular cargos, nanobody‐CPP chimeras also enabled degradation of extracellular proteins, exemplified by the ovarian tumor marker—human epididymis protein 4 (HE4), resulting in effective suppression of ovarian cancer cell proliferation and migration. Furthermore, a PTD‐enhanced endobody targeting EGFR successfully degraded the receptor on lung cancer cell surfaces, inhibited cell proliferation, and suppressed tumor growth in a lung cancer xenograft mouse model.

During the course of manuscript submission and subsequent to the related patent application (filed in 2024), another TPD strategy termed CPPTAC was recently reported [37], which features a small molecule binder conjugated with a CPP capable of inducing membrane protein degradation in a receptor‐independent manner. In comparison, endobodies are not only genetically encodable facilitating preparation, but is also capable of targeting undruggable proteins. In our work, we showcased the targeted degradation of human epididymis protein 4 (HE4), an undruggable glycoprotein free of stable binding pockets using a HE4‐specific endobody. Additionally, by taking the advantage of the genetically encodable feature, we created a bispecific endobody, enabling simultaneous degradation of both an extracellular and a membrane protein. This offers new opportunities for creating advanced degraders exhibiting synergistic pharmacological effects.

For this bispecific endobody, all modules are connected by a (GGS)_2_ flexible linker, which allows correct folding of NbHE4 and NbEGFR and avoids potential steric hindrance that may disrupt target binding. While the exact epitopes targeted by NbHE4 remain unknown, functional degradation assays (Figure 4J) confirm that this linker design is sufficient to achieve simultaneous degradation of both HE4 and EGFR.

There are also some other MPDs which feature a CPP element, such as GlueTAC [12], Pro‐MPD [38], PSMLTACs [39], and very recently the reported iVAC [40]. However, these modalities require extra essential components such as encoded UAAs for covalent binding, lysosomal sorting sequences, or biparatopic nanobodies to achieve effective degradation.

In this work, we demonstrate that a conventional CPP fused to a nanobody is sufficient to drive efficient membrane protein degradation, establishing a foundation for designing more advanced MPDs. Through systematic evaluation of cationic, amphiphathic, and hydrophobic CPPs, we show that nanobody‐CPP fusion represents a generalizable strategy for creating structurally‐concise degraders. Furthermore, incorporating a proteasome‐targeting domain enhanced degradation potency via a dual‐mechanism pathway. Collectively, nanobody‐CPP chimeras, or endobodies, represent a versatile and genetically encodable class of MPDs with broad utility in both basic research and therapeutic applications.

## Author Contributions

**Chengjian Zhou**: conceptualization, methodology, data curation, investigation, validation, formal analysis, visualization, writing – review and editing. **Xi Chen**: conceptualization, methodology, data curation, investigation, validation, formal analysis, supervision, funding acquisition, visualization, project administration, resources, writing – original draft, writing – review and editing. **Huiping He**: methodology, data curation, investigation, visualization. **Simin Xia**: methodology, data curation, investigation, visualization.

## Ethical Approval for Animal Experiments

All animal experiments were approved by the Institutional Animal Care and Use Committee of Harbin Institute of Technology (approval No. IACUC‑2021052).

## Conflicts of Interest

This work has been filed for Chinese invention patent applications (Application Nos. 202411681769.4 and 202610723866.8).

## Supporting information




**Supporting File 1**: advs76075‐sup‐0001‐SuppMat.docx.

**Supporting File 2**: advs76075‐sup‐0002‐DataFile.xlsx.

## Data Availability

The data that supports the findings of this study are available in the supplementary material of this article.
