# Supplementary material for: Endobody: Genetically Encodable Nanobody‐CPP Chimeras for Degradation of Membrane and Extracellular Proteins
Source: Adv Sci (Weinh). 2026 Jun 23;13(50):e76075. doi: 10.1002/advs.76075 (PMC13336368; doi:10.1002/advs.76075)
Supplement: Supplementary file 1 [file ADVS-13-e76075-s001.docx]

**Supplementary Information**

**Endobody:** Genetically Encodable Nanobody-CPP Chimeras for Degradation of Membrane and Extracellular Proteins

Chengjian Zhou^1,2,‡^, Huiping He^1,2,‡^, Simin Xia^1^, Xi Chen^1,2,^*

^1^ Laboratory of Chemical Biology and Frontier Biotechnologies, The HIT Center for Life Sciences, Harbin Institute of Technology, Harbin 150001, P. R. China

^2^ Faculty of Life Sciences and Medicine, Harbin Institute of Technology, Harbin 150001, P. R. China

^‡^ Equal contribution

* Lead contact: [chenxihit@hit.edu.cn](mailto:chenxihit@hit.edu.cn)

**Methods**

**Animal welfare**

Regarding mice, animals were maintained under specific pathogen-free (SPF) conditions and handled based on the approval by the Institutional Animal Care and Use Committee of Harbin Institute of Technology (IACUC/HIT) with the permit number IACUC-2021052. Mice were housed under controlled light (12 h light/12 h dark cycle), temperature (24 ± 2 °C) and humidity (50 ± 10%) conditions and fed a normal chow diet with a free access to water. As per ARRIVE guidelines tumor sizes did not exceed 10% of the animal body weight and tumor volumes did not exceed 2000 mm^3^ (20 mm in diameter). As per guidelines, animal weight loss did not exceed 15% of the original body weight from the start of the study, throughout xenograft model generation and subsequent drug administration (Day −12 or -10 to Day 15).

**Generation of xenograft mouse model and drug treatment**

Female BALB/c nude mice (4-6 weeks old, RRID: IMSR_CRL: 194) were purchased from Charles River (Beijing). A549 cells from Procell Life Science & Technology (#CL-0016, RRID: CVCL_0023) were grown in a standard Φ ~ 85mm Petri dish to exponential phase before harvesting. Then A549 cells were first washed by 10 ml PBS, added 1 ml of trypsin, digested for 2-3 min to allow full detachment of cells from the growing surface, and added 3 ml of full cell culture medium to suspend the detached cells. The cell suspension was subjected to centrifugation at 1000 g for 5 min at 4 °C. The clear supernatant was discarded and the cell pellets were resuspended in a freshly prepared ice-cold 1:1 (v/v) mixture of PBS/Matrigel (Cat# M8370, Solarbio) on ice. The final cell density is ~5×10^7^ ml^-1^. To make A549 xenograft mouse model, ~6×10^6^ A549 cells in 0.1 ml of PBS/Matrigel solution were subcutaneously injected into the axillary region of the BALB/c nude mice. Tumor will typically appear in 1-2 weeks and will continue to grow steadily. In order to evaluate the efficacy of endobodies, EER_9_P1 (10 mg∙kg^-1^), 2×NbEGFR (10 mg∙kg^-1^), or PBS alone were intratumorally injected to the mice and the injection was repeated every two days. Tumor sizes were monitored every day using a Vernier calipers. Tumor volumes were calculated based on the following equation: V = (length × width^2^)/2.

**Mammalian cell culture**

HeLa (#CL-0101, RRID: CVCL_0030), A549 (#CL-0016, RRID: CVCL_0023), MDA-MB-231(#CL-0150, RRID: CVCL_0062), OVCAR3 (#CL-0178, RRID: CVCL_0465), HEK-293T (#CL-0005, RRID: CVCL_0063), and SK-BR-3 (#CL-0211, RRID: CVCL_0033) cells were obtained from Procell Life Science & Technology Co., Ltd. (Wuhan, P.R. China). The cells lines were short tandem repeat (STR) identified and proven to be HIV-1, HBV, HCV, mycoplasma, and other microorganisms free before culturing. Other reagents such as full DMEM (Dulbecco's modified Eagle's medium) and PBS (phosphate buffered saline) were also confirmed to be mycoplasma free before usage. All cell cultures were maintained at 37 °C under 5 % CO_2_ in high glucose (4.5 g∙L^−1^) DMEM (HyClone, #SH30243.01) containing 4 mM L-glutamine and sodium pyruvate and supplemented with additional 10 % fetal bovine serum (FBS) (HyClone, #SV30087.03), 1 % non-essential amino acid (NEAA, 100×), and 1 % penicillin-streptomycin (100×). Trypsin-EDTA (HyClone, #SH30042.01) and PBS (HyClone, #SH30256.01) were used in subculturing. All cell cultures were subcultivated in a ratio of 1:5~10.

**Plasmid construction**

Plasmid vectors, such as pTXB1, EGFP-C1 and EGFP-N1 were obtained from commercial vendors. These parental vectors may be further engineered, such as introducing a His_6_ tag, alternation of restriction cleavage sits, or replacing EGFP by other fluorescent proteins, e.g. mCherry, etc. to give modified versions of the parental vector for cloning. Subcloning, Gibson cloning, or modified Gibson cloning methods were employed to construct the desired plasmids. For subcloning, fragments of interest were directly cut from the parent plasmid using appropriate restriction enzymes, or amplified by PCR from plasmids containing the desired genes using hyPerFUsion high-fidelity polymerase (APExBIO, #1032,), gel purified, digested with restriction enzymes and purified again. Multiple fragments were assembled by stepwise subcloning or one-step multi-fragment Gibson cloning. Genes of interest were obtained *via* custom gene synthesis from Comate Bioscience Co., Ltd. (Changchun, P.R. China) or Ruibiotech (Beijing, P.R.China). Alternatively, plasmids containing the desired genes can be purchased from Miaoling Plasmid Sharing Platform if applicable.

**Transfection**

Transient transfection was typically performed in an 8-well (#155409) Lab-Tek®II imaging chamber from Thermo Scientific using Lipo8000^TM^ transfection reagent from Beyotime Biotechnology (#C0533). Typically, 0.25 μg DNA was dissolved in 12.5 µl gibco opti-MEM (Life technologies, #31985-062) and then 0.4 μl Lipo8000^TM^ transfection reagent was added and mixed *via* gentle pipetting. Then this mixture was added into an imaging chamber well seeded with 2.5×10^4^ cells that were already adhesively attached on the bottom in 250 μl full DMEM. The cells were maintained under 5% CO_2_ at 37 °C for around 2 h. Then the medium was replaced by warm full DMEM and the cells were further incubated under 5% CO_2_ at 37 °C for over 20 h. For co-transfection of more than one plasmid, the quantity of DNA used in this protocol implies the total amount of plasmids.

**Confocal microscopy**

Live cells were imaged in phenol red free Dulbecco‘s Modified Eagle Medium (Life Technologies, #21063-29) supplemented with additional 10 % FBS, 1 % sodium pyruvate, 1 % NEAA, 1 % penicillin-streptomycin and 15 mM HEPES-Na at 37 °C under 5 % CO_2_. Microscopy was performed using Nikon A1 ECLIPSE T*i*2 inverted confocal microscope. The microscope was equipped with four lasers (405 nm, 488 nm, 561 nm, and 640 nm), allowing the acquisition of confocal fluorescence data for four different excitation wavelengths. For detection of blue (excited by 405 nm laser) or far red (excited by 640 nm) fluorescence signal, PMT (photomultiplier tube) detectors were used; while for the detection of green (excited by 488 nm) or red (excited by 561 nm) fluorescence signal, the more advanced GaAsP detector with even higher sensitivity will be used. Most images presented in this article were acquired with a 60× oil objective lens (APO 60×/1.4 oil) having a numerical aperture of 1.4. Alternatively, a 40× objective lens (Plan Apo 40×/0.95) having a numerical aperture of 0.95 can be used, for example when capturing images with larger field of views. In most cases, the basic imaging setup were configured with typical parameters set as follows: Scan speed 0.5, number of averaging 4, scan line mode in one-way scan direction.

**Protein expression and purification**

***General protocol***

pTXB1 vector was used to express intein-tag fused nanobody chimeras for expressed protein ligation (EPL). These plasmids for protein expression were first transformed into *E. coli* Rosetta 2a cells and the transformants were selected on ampicillin (100 mg∙L^−1^) or kanamycin (50 mg∙L^−1^) agar plates depending on the antibiotic resistance of the plasmids. A single colony was used to inoculate 50-100 ml of LB medium containing 100 mg∙L^−1^ ampicillin or 50 mg∙L^−1^ kanamycin and shaken at 240 rpm for 8-10 hours or overnight at 37 °C. 30-50 ml of the preculture was used to further inoculate ~1.8 L fresh LB medium containing 100 mg∙L^−1^ ampicillin or 50 mg∙L^−1^ kanamycin, and additional chloramphenicol (33 mg∙L^−1^). The absorbance at 600 nm (OD600) of the inoculated culture should be controlled between 0.05 to 0.1 in this inoculation step. Then the culture was shaken at 180 rpm at 37 °C for a few hours (typically 2-3 h) until OD 600 reached 0.5-0.6. Then 0.5 ml isopropyl β-D-thiogalactoside (IPTG) stock solution (1 M) was added (final ~0.27 mM) to induce protein expression at 37 °C for 5 h, or at 16 °C overnight. Sometimes protein expression time and temperature needed to be optimized in order to achieve an optimal expression for some particular proteins.

Later, cells were harvested by centrifugation at 13881× g, at 4 °C for 15 min and washed once with PBS (4149× g, 10 min). The bacterial pellet was resuspended in lysis buffer (pH 8.0, PBS supplemented with additional 0.5 M NaCl, 3 % glycerol, w/o 3 mM β-mercaptoethanol (BME), 1 mM phenylmethylsulfonyl fluoride (PMSF). For relatively smaller volumes of bacterial cell suspensions (< 40 ml), bacterial cells were typically lysed *via* ultra-sonification at 80 W for 30 min or 60 W for 45 min (1 s sonification followed by 3 s interval) on ice. For batch processing or larger volumes of cell suspensions, cells were typically lysed using ultra-high-pressure homogenizer cooled by a bench chiller for 2-3 cycles under 800-900 bar at 4 °C. The lysate was cleared by high-speed centrifugation (74766× g, 45 min, 4 °C) and the supernatant was loaded onto a gravity Ni-NTA column (2-5 ml resin). The Ni-NTA column was washed and then the His-tag fused protein was eluted using step-gradient of imidazole (50, 100, …, until 500 mM) solutions. Alternatively, GE ÄKTA Pure machine equipped with a HisTrap FF column was used to purify His-tagged protein *via* gradient elution (0 🡪 500 mM imidazole) by combining buffer A (pH 8.0 PBS, 0.5 M NaCl, 3 % glycerol, w/o 3 mM BME) and buffer B (pH 8.0 PBS, 0.5 M imidazole, 0.5 M NaCl, 3 % glycerol, w/o 3 mM BEM). Ionic exchange or size exclusion chromatography may be further applied if additional purifications are necessary. The obtained proteins were typically concentrated, buffer exchanged in buffer A, aliquoted, snap frozen in liquid nitrogen, and stored under -80 °C.

***Preparation of endobodies or nanobodies via expressed protein ligation***

Proteins to be ligated were expressed as a fusion chimera with a *C*-terminal Mxe GyrA intein tag by cloning the respective gene into an optimized pTXB1 vector (Conjubody Biotek, #NB-001; [www.snacip.com](http://www.snacip.com)) demonstrating improved ligation yield. The use of the pTXB1 vector contains an intein tag will generally increase the bacterial expression yield of endobodies. Afterwards, this fusion chimera was expressed, purified and buffer exchanged in Buffer A (pH 8.0 PBS, 0.5 M NaCl, 3 % glycerol). Typically, the Mxe GyrA intein fusion protein was reconstituted to around 15 mg∙mL^−1^ before ligation. To initialize the ligation reaction, 1/2 volume of 2 M sodium 2-mercaptoethanesulfonate (MENSNa) stock solution (Conjubody Biotek, #NB-002) was added as the intein cleavage reagent and 1/2 volume of 1.1 M 4-mercaptophenylacetic acid (MPAA) stock solution (Conjubody Biotek, #NB-003) was added as the catalyst. Finally, L-cysteine (66 mM/H_2_O) was added to the reaction solution at a final concentration of 2 mM. The reaction mixture was incubated at 4 °C for 3 days to achieve conversion typically over 90 %. The ligated product was then purified *via* step-gradient (0🡪500 mM imidazole) reverse gravity Ni-IMAC chromatography using high affinity Ni-charged Resin FF (GenScript, #L00666-25,). In this process, cleaved intein-His_6_ fragment will bind onto the resin and pure nanobody chimeras will be eluted out.

***Preparation of NbEGFR-R_9_ endobody*** *(example 1)*

In a typical preparation, the reaction mixture was prepared by sequentially adding 0.5 ml of MPAA (1.1 M) and 0.5 ml of MENSNa (2 M) to 1 ml of NbEGFR-R_9_-intein-His_6_ solution (20 mg∙ml^-1^ in buffer A). 60 µl L-cysteine (66 mM stock) was then introduced to achieve a final concentration of 2 mM. The mixture was centrifuged at 13,500 rpm for 1 min at 4 °C. Following brief sonication, the solution was added argon to establish an anaerobic environment. The reaction vial was securely sealed under argon atmosphere and incubated at 4 °C for 3 days.

Conversion will reach maximal after a few days as monitored by SDS-PAGE, and the reaction was subjected to Ni-NTA IMAC purification to remove cleaved intein-His_6_ and some unreacted NbEGFR-R_9_-Intein-His_6_. Otherwise, SEC can be used for the separation. The purified protein was concentrated and buffer-exchanged into buffer A using a 10 kDa centrifugal ultrafiltration device, yielding a final concentration of 5.6 mg∙ml^-1^ with a total volume of 0.5 ml.

***Preparation of NbPD-L1-R_9_ endobody*** *(example 2)*

In a typical preparation, the reaction mixture was prepared by sequentially adding 0.5 ml of MPAA (1.1 M) and 0.5 ml of MENSNa (2 M) to 1 ml of NbPD-L1-R_9_-intein-His_6_ solution (20 mg∙ml^-1^ in buffer A). 60 µl L-cysteine (66 mM stock) was then introduced to achieve a final concentration of 2 mM. The mixture was centrifuged at 13,500 rpm for 1 min at 4 °C. Following brief sonication, the solution was added argon to establish an anaerobic environment. The reaction vial was securely sealed under argon atmosphere and incubated at 4 °C for 3 days.

Conversion will reach maximal after a few days as monitored by SDS-PAGE, and the reaction was subjected to Ni-NTA IMAC purification to remove cleaved intein-His_6_ and remained unreacted NbPD-L1-R_9_-Intein-His_6_. The purified protein was concentrated and buffer-exchanged into buffer A using a 10 kDa centrifugal ultrafiltration device, yielding a final concentration of 1.9 mg∙ml^-1^ with a total volume of 1.1 ml.

***Preparation of NbHER2-R_9_ endobody*** *(example 3)*

In a typical preparation, the reaction mixture was prepared by sequentially adding 0.15 ml of MPAA (1.1 M) and 0.15 ml of MENSNa (2 M) to 0.3 ml of NbHER2-R_9_-intein-His_6_ solution (20 mg∙ml^-1^ in buffer A). 18.2 µl L-cysteine (66 mM stock) was then introduced to achieve a final concentration of 2 mM. The mixture was centrifuged at 13,500 rpm for 1 min at 4 °C. Following brief sonication, the solution was added argon to establish an anaerobic environment. The reaction vial was securely sealed under argon atmosphere and incubated at 4 °C for 3 days.

Conversion will reach maximal after a few days as monitored by SDS-PAGE, and the reaction was subjected to Ni-NTA IMAC purification to remove cleaved intein-His_6_ and remained unreacted NbHER2-R_9_-Intein-His_6_. The purified protein was concentrated and buffer-exchanged into buffer A using a 10 kDa centrifugal ultrafiltration device, yielding a final concentration of 4.1 mg∙ml^-1^ with a total volume of 0.35 ml.

***Preparation of NbHE4-NbEGFR-R_9_ bispecific endobody*** *(example 4)*

In a typical preparation, the reaction mixture was prepared by sequentially adding 0.15 ml of MPAA (1.1 M) and 0.15 ml of MENSNa (2 M) to 0.3 ml of NbHE4-NbEGFR-R_9_-intein-His_6_ solution (20.2 mg∙ml^-1^ in buffer A). 18 µl L-cysteine (66 mM stock) was then introduced to achieve a final concentration of 2 mM. The mixture was centrifuged at 13,500 rpm for 1 min at 4 °C. Following brief sonication, the solution was flushed with argon to establish an anaerobic environment. The reaction vial was securely sealed under argon atmosphere and incubated at 4 °C for 3 days.

Reaction conversion will reach maximal after a few days as monitored by SDS-PAGE, and the reaction was subjected to Ni-NTA IMAC purification to remove cleaved intein-His_6_ and remained unreacted NbHE4-NbEGFR-R_9_-Intein-His_6_. The purified protein was concentrated and buffer-exchanged into buffer A using a 10 kDa centrifugal ultrafiltration tube, yielding a final concentration of 4.2 mg∙ml^-1^ with a total volume of 0.3 ml.

***Preparation of 2×NbEGFR-R_9_-PTD1 (EER_9_P1) endobody*** *(example 5)*

In a typical preparation, the reaction mixture was prepared by sequentially adding 0.4 ml of MPAA (1.1 M) and 0.4 ml of MENSNa (2M) to 0.8 ml of NbEGFR-R_9_-intein-His_6_ solution (15 mg∙ml^-1^ in buffer A). 48 µl L-cysteine (66 mM stock) was then introduced to achieve a final concentration of 2 mM. The mixture was centrifuged at 13,500 rpm for 1 min at 4 °C. Following brief sonication, the solution was added argon to establish an anaerobic environment. The reaction vial was securely sealed under argon atmosphere and incubated at 4 °C for 3 days.

Conversion will reach maximal after a few days as monitored by SDS-PAGE, and the reaction was subjected to Ni-NTA IMAC purification to remove cleaved intein-His_6_. Otherwise, SEC can be used for the separation. The purified protein was concentrated and buffer-exchanged into buffer A using a 10 kDa centrifugal ultrafiltration device, yielding a final concentration of 3.2 mg∙ml^-1^ with a total volume of 0.8 ml.

**SDS-PAGE and in-gel fluorescence**

Endobodies, nanobodies, and their EPL reactions were analyzed by SDS–PAGE. A 15% or 12% polyacrylamide gel was prepared using a commercial kit (Genefist, #GF1820-15) following the manufacturer’s protocol. Protein samples were quantified, mixed with 5× loading buffer (Biosharp, #BL511A), boiled at 90 °C for 5 min, and briefly centrifuged before loading. Electrophoresis was conducted in 1× pH 8.3 Tris-glycine running buffer (25 mM Tris, 192 mM glycine, 0.1% SDS) at 180 V for ~50 min until the dye front reached the gel bottom. A pre-stained protein ladder (10–180 kDa; Genefist, #GF6610-10) served as the molecular weight marker.

**Western blot (WB) analysis**

Cells were plated in 24-well plate at a density of 6x10^4^ cells per well, and then left to grow overnight to allow cell adhesion before drug treatment at the specified concentrations. For the degradation of overexpressed protein such as EGFP and EGFP-fused proteins, cells were first subjected to transfection using respective vectors before adding drugs. Unless otherwise specified, after 24 hours or specific time, cells were washed (2 × PBS), collected using 2× SDS-PAGE loading buffer (120 μl/well for 24-well plates), and then boiled at 90 °C for 5 minutes before subsequent gel electrophoresis and WB analysis.

For WB analysis, cell lysate samples were first subjected to 12% SDS-PAGE gel electrophoresis (180 V, 55 min), transferred to nitrocellulose (NC) membrane (PALL, #66485) on ice applying constant 400 mA current for 30 min in rapid WB transferring buffer (Genefist, #GF1816). The NC membrane was blocked using 5 % skim milk (Biosharp, #BS102) in 1× TBST at room temperature for 1.5 h. Then, the blocked NC membrane was labeled by primary antibodies diluted in 5 % skim milk at 4 °C overnight.

These primary antibodies include: GFP-tag rabbit mAb antibody (Zenbio, #R24437, RRID: AB_3751046) diluted at a ratio of 1:1000, GAPDH pAb antibody (Bioworld, #AP0063, RRID: AB_2651132) diluted at a ratio of 1:5000, EGFR rabbit mAb antibody (Zenbio, #R22778, RRID: AB_3719349) diluted at a ratio of 1:1000, PD-L1 rabbit mAb antibody (Zenbio, #R30023, RRID: AB_3751047) diluted at a ratio of 1:1000, HER2 rabbit mAb antibody (Zenbio, #R380836, RRID: AB_3751048) diluted at a ratio of 1:1000, HE4 rabbit mAb antibody (Zenbio, #R381649, RRID:AB_3751049) diluted at a ratio of 1:1000, β-actin rabbit mAb antibody (ABclonal, #AC026, RRID: AB_2768234) diluted at a ratio of 1:50000, AKT1/2/3 rabbit mAb antibody (Zenbio, #R23412, RRID: AB_3751050) diluted at a ratio of 1:1000, phospho-AKT1 (Ser473) rabbit mAb antibody (Zenbio, #R381555, RRID: AB_3751051) diluted at a ratio of 1:1000, ERK1/2 rabbit pAb antibody (Zenbio, #343830, RRID: AB_3073887) diluted at a ratio of 1:1000 and phospho-ERK1/2 (Thr202/Tyr204)/(Thr185/Tyr187) rabbit mAb antibody (Zenbio, #R24245, RRID: AB_3076564) diluted at a ratio of 1:1000.

The next day, NC membrane was washed by TBST (3×5min), incubated with secondary antibody using HRP-conjugated goat anti-rabbit IgG antibody (Zenbio, #511203, RRID: AB_2927753) diluted in 5 % skim milk at a ratio of 1: 5000 at room temperature for 1h, and washed by TBST (3×10min) before imaging. For WB signal detection, the membrane was developed with a mixture of high-sensitivity luminescent liquid (Biosharp, #BL523B). Densitometry of developed bands was measured and analyzed using LI-COR Odyssey Fc imaging system.

**Immunoprecipitation (IP) assay**

For immunoprecipitation assays, HeLa cells were lysed in RIPA (radio immunoprecipitation assay) lysis buffer (Beyotime, #P0013B). Aliquots of 150 μL of cell lysate containing equal amounts of total protein were incubated with NbEGFR or NbEGFR‑R_9_ at a final concentration of 2 μM at 4 °C for 2 h. Meanwhile, anti‑V_HH_ antibody (abinScience, #ZY145013, RRID: AB_3751045) was conjugated to protein A/G magnetic beads (Yeasen, #36427ES) at room temperature for 1 h. The beads were then washed three times with 1×TBST and subsequently mixed with the nanobody‑treated cell lysate. The mixture was incubated at 4 °C overnight with gentle rotation. The next day, the beads were washed three times with 1×TBST, and bound proteins were eluted by boiling in SDS loading buffer, followed by Western blot analysis using anti-EGFR antibody and anti‑V_HH_ antibody.

**Fluorescent labeling of endobodies**

Endobody chimeras were fluorescently labeled using either 5-FAM-NHS (5-carboxyfluorescein N-hydroxysuccinimide ester) or 5-TMR-NHS (5-carboxyltetramethylrhodamine N-hydroxysuccinimide ester). The labeling reaction was carried out by mixing the endobody chimeras with the respective NHS-ester dye at a molar ratio of 1:1 (protein:dye) in PBS (pH 7.4). The mixture was gently vortexed and then incubated at 4 °C overnight. To quench the remaining reactive NHS-ester groups, Tris-HCl (pH 8.0) was added to the reaction mixture at a final concentration of 20 mM, followed by incubation at room temperature for 30 min. Owing to the negative charge of the fluorescent dyes and the lack of conjugated CPPs, the small fraction of quenched dyes cannot penetrate the cell membrane, thus will not affect the visualization of the cellular uptake of endobodies. The resulting fluorescently labeled endobody chimeras were either used directly or stored at 4 °C in dark for further experiments.

**CCK-8 cell viability assay**

The cytotoxic effects of R_9_ and GBP-R_9_ were evaluated using the Cell Counting Kit-8 (CCK-8; Beyotime Biotechnology, China, #C0037) in five human cancer cell lines: HeLa, MDA-MB-231, SK-BR-3, OVCAR3, and A549. Cells in the logarithmic growth phase were seeded into 96-well plates at a density of 5×10^3^ cells per well in 100 μL of culture medium and incubated overnight to allow cell adhesion. The next day, culture medium was replaced with fresh medium containing serial dilutions of R_9_ or GBP-R_9_ at final concentrations of 0, 0.05, 0.1, 0.25, 0.5, 1, 2.5, 5, 10, 25, 50, and 100 μM. The 0 μM control received an equal volume of PBS. Each concentration was tested in triplicate wells. After 24 h of treatment at 37 °C, 10 μL of CCK-8 solution was added to each well, and the plates were incubated for an additional 1 h under the same conditions. The absorbance at 450 nm was measured using a microplate reader. The absorbance of blank wells (medium without cells) was subtracted from all readings. Cell viability (%) was calculated as (absorbance of treated wells / absorbance of control wells) × 100%.

**EdU cell proliferation assay**

The EdU cytotoxicity/ cell proliferation assay was performed using an EdU cell proliferation detection kit (RiboBio, #R11053.9). Briefly, 2.5×10^4^ HeLa cells harvested at exponential phase were seeded in an 8-well (#155409) Lab-Tek®II imaging chamber from Thermo Scientific suitable for high resolution confocal imaging. Afterwards, cells were allowed to grow overnight. Drug solutions at specified final concentrations in full DMEM was used to treat the cells. After a given incubation time, the drug solution was replaced by EdU solution at a final concentration of 50 μM. It was incubated for 2 hours at 37 °C under 5% CO_2_ as recommended for general cancer cell lines. Then, each well was washed by PBS (2×5 min) to remove the excess of EdU, added with 100 μl of fixative solution (4% PMA in PBS) and incubated for 30 min at room temperature. Then 100 μl of 2 mg∙ml^−1^ glycine solution was added to each well and shaken at RT for 5 min to quench the fixative. Glycine solution was removed and each well was washed by 200 μl PBS and shaken at RT for 5 min. PBS was removed and each well was added with 200 μl cell permeabilization solution (0.5% TritonX-100 in PBS) and shaken at RT for 10 min. The fixed cells were further washed by PBS (1×5 min) before labeling.

Before fluorescent labeling by click reaction, 1× Apollo labeling solution that contains the red color Apollo567 dye (RiboBio, #C10310-1), catalyst and other necessary reagents were freshly prepared according to the manufacture’s guidance. For example, 1 ml of 1× Apollo labeling solution could be prepared by sequentially adding 938 μl DI-H_2_O, 50 μl Apollo reaction buffer (reagent B), 10 μl Apollo catalyst solution (Cu^2+^, buffer C), 3 μl Apollo 567 dye (reagent D) and ~9 mg Apollo additive (sodium ascorbate, reagent E). 200 μl freshly prepared 1× Apollo labeling solution was added into each well, shielding from light, and shaken at RT for 30 min to complete the click labeling. Labeling solution was removed, and the cells in each well were washed by permeabilization solution (0.5% TritonX-100 in PBS) again (3×10min). Permeabilization solution was removed and the cells were wash by PBS (1×5 min). Finally fresh PBS was added and the labeled cells were ready for confocal microscopy imaging. Hoechst could be used to label the nucleus if necessary.

**Hematoxylin-eosin (HE) staining**

Tumor tissues and major organs were fixed in 4% paraformaldehyde at 4 °C temperature for 12 h, followed by dehydration, permeabilization, embedding and sectioning. After dewaxing and antigen retrieval, the sections were stained by hematoxylin-eosins followed by dehydration and mounting. The stained sections were observed and photographed under a light microscope.

**Image analysis**

Microscopic images were analyzed and processed with ImageJ/Fiji and prepared for presentation using Microsoft Office PowerPoint. Image manipulations were restricted to adjustment of brightness level (i.e. linear stretch), background subtraction, cropping, rotating, scaling, and false color-coding using Look-Up Tables (LUT).

**Statistics and reproducibility.**

All microscopic imaging experiments were representative of at least three independent repeats if not otherwise stated; representative SDS-PAGE images were from at least three independent repeats with similar results; representative confocal microscopic images were from at least ten independent cells with similar results. No randomization nor blinding was used in this study. Graphpad and Microsoft Excel were used for plotting, data fitting, graphing and statistical analysis. Student’s *t*-tests were used to compare two experimental conditions. Unless otherwise specified, one-sided unpaired *t*-tests were performed, as for example cells with or without drug treatment. When necessary, stars were used to denote *P*-values for indicated statistical tests (*: *P*<0.05; **: *P*<0.01; ***: *P*<0.001; ****: *P*<0.0001). Exact *P*-values were indicated for critical experiments.

**Supplementary Table**

| Nanobody | Affinity (Kd) | Binding Epitope / Domain | Key Binding Residues (on antigen) | Pharmacological Activity (Classification) |
| --- | --- | --- | --- | --- |
| **GBP** | 1.4 nM | Conformational epitope on GFP, located at the C-terminal side of the β-barrel, mainly involving β-strands 8-11 | Leu221, Ala206, Phe223 (hydrophobic); Arg168, Glu142 (salt bridge); Asn146 (hydrogen bond, specificity determinant); Val176, Ser175 | Neutral binder (no intrinsic signaling activation or blocking activity) |
| **NbEGFR** | 219 ± 20 nM | Flat surface on Domain III of EGFR, partially overlapping with the EGF binding site | D355, F357, R353, Q384 | Ligand-competitive antagonist (prevents ligand-induced EGFR activation) |
| **NbPD-L1** | 3.0 nM | N-terminal immunoglobulin variable-like domain of human PD-L1 (residues 19-132) | Tyr56, Ile54, Gln66, Glu58, Arg113 | Ligand-competitive antagonist (competes with PD-1 for binding to PD-L1) |
| **NbHER2** | 3.99 ± 0.04 nM | Domain I of HER2 (residues Gln2-Arg196) | Not specified | Neutral binder (does not directly agonize or antagonize HER2 signaling, nor block ligand binding) |
| **NbHE4** | 1.6 nM | Not specified (no X-ray crystallography or mutagenesis mapping reported in the literature) | Not specified | Antagonist; neutralizes secreted autocrine HE4 protein |

**Supplementary Table 1:** List of used nanobodies in the manuscript with their characteristics and related information noted.

**Supplementary Figures**

**Supplementary Figure 1**. Preparation of NbEGFR-CPP chimeras bearing different CPPs through expressed protein ligation (EPL) with free L-Cysteine. (A) Bioconjugation scheme for the preparation of NbEGFR-CPP chimeras. (B) Denaturing SDS-PAGE (15%) characterization of EPL reaction for 3 days and the pure NbEGFR-R_9_ chimera. (C) Denaturing SDS-PAGE (15%) characterization of EPL reaction for 3 days and the pure NbEGFR-TAT chimera. (D) Denaturing SDS-PAGE (15%) characterization of EPL reaction for 3 days and the pure NbEGFR-pVEC chimera. (E) Denaturing SDS-PAGE (15%) characterization of EPL reaction for 3 days and the pure NbEGFR-Bac7 chimera. (F) Denaturing SDS-PAGE (15%) characterization of EPL reaction for 3 days and the pure NbEGFR-C105Y chimera. (G) Denaturing SDS-PAGE (15%) characterization of EPL reaction for 3 days and the pure NbEGFR-Pep7 chimera.

**Supplementary Figure 2.** NbEGFR-R_9_ chimera reveals the highest colocalization with early endosomes. (A) Representative fluorescence micrographs of HeLa cells treated with 5-FAM-labeled NbEGFR-CPP chimeras (1 μM, 24 h, 2×PBS washout) revealed colocalization with early endosomes visualized by mCherry-Rab5a. Arrows denote the potentially escaped fraction of FAM‑labeled NbEGFR‑CPP chimeras (green signal). Scale bars: 10 μm. (B) Pearson’s correlation coefficient (PCC) analysis between 5-FAM-labeled NbEGFR-CPPs with early endosomes (n=10 cells). (C) CCK-8 assay for the evaluation of the cytotoxicity of R_9_ at different concentrations across different cell types (24 h; n = 3 independent experiments). (D) CCK-8 assay for the evaluation of the cytotoxicity of GBP-R_9_ at different concentrations across different cell types (24 h; n = 3 independent experiments).

**Supplementary Figure 3.** Preparation and evaluation of NbEGFR as a non-functional control for NbEGFR-R_9_ endobody as well as the evaluation of the impact of R_9_ moiety on antigen binding and the presence of free R_9_ on protein degradation. (A) Denaturing SDS-PAGE (15%) characterization of the EPL reaction revealing near-full conversion of NbEGFR-intein-His_6_ precursor (left) and the pure NbEGFR chimera after reverse Ni-IMAC separation (right). (B) WB analysis revealed that NbEGFR could not trigger detectable degradation of EGFR up to 20 μM concentration. n = 3 experiments. One-sided independent Student’s *t*-text used. (C) Fusion of R_9_ moiety does not affect antigen binding capacity of NbEGFR according to immunoprecipitation experimental result. (D) The presence of free R_9_ peptide (10 μM) does not affect the degradation of EGFR by NbEGFR-R_9_ endobody (10 μM, 24 h). n = 3 experiments. One-sided independent Student’s *t*-text used.

**Supplementary Figure 4.** Preparation of NbPD-L1-R_9_ endobody and NbPD-L1 (control), and functional evaluation of NbPD-L1. (A) Denaturing SDS-PAGE (15%) characterization of the EPL reaction revealing near-full conversion of NbPD-L1-R_9_-intein-His_6_ precursor (left) and the pure NbPD-L1-R_9_ chimera after reverse Ni-IMAC separation (right). (B) Denaturing SDS-PAGE (15%) characterization of the EPL reaction revealing near-full conversion of NbPD-L1-intein-His_6_ precursor (left) and the pure NbPD-L1 chimera after reverse Ni-IMAC separation (right). (C) WB analysis revealed that NbPD-L1 could not trigger detectable degradation of PD-L1 in live MDA-MB-231 breast cancer cells up to 20 μM concentration. n = 3 experiments. One-sided independent Student’s *t*-text used.

**Supplementary Figure 5.** Preparation of NbHER2-R_9_ endobody and Nb HER2 (control), and functional evaluation of NbHER2. (A) Denaturing SDS-PAGE (15%) characterization of the EPL reaction revealing near-full conversion of NbHER2-R_9_-intein-His_6_ precursor (left) and the pure NbHER2-R_9_ chimera after reverse Ni-IMAC separation (right). (B) Denaturing SDS-PAGE (15%) characterization of the EPL reaction revealing near-full conversion of NbHER2-intein-His_6_ precursor (left) and the pure NbHER2 chimera after reverse Ni-IMAC separation (right). (C) WB analysis revealed that NbHER2 could not trigger detectable degradation of HER2 in live SK-BR-3 breast cancer cells up to 20 μM concentration. n = 3 experiments. One-sided independent Student’s *t*-text used. (D-E) NbEGFR-R_9_ endobody, rather than control GBP-R_9_ endobody, induces EGFR degradation in EGFR/PD-L1 double-positive MDA-MB-231 cells, while neither construct alters the level of another membrane protein PD-L1, verifying that endobodies specifically degrade their recognized membrane protein targets without affecting unrelated membrane proteins (n = 3 independent experiments, one-sided unpaired Student’s *t*-test, mean ± SD).

**Supplementary Figure 6.** Preparation of NbHE4-R_9_ endobody and NbHE4 as the negative control. (A) Denaturing SDS-PAGE characterization of the EPL reaction and the purified NbHE4-R_9_ endobody. (B) Denaturing SDS-PAGE characterization of the EPL reaction and the purified NbHE4 nanobody.

**Supplementary Figure 7.** Preparation of NbHE4-NbEGFR-R_9_ bispecific endobody for simultaneous extracellular and membrane protein degradation. (A) Denaturing SDS-PAGE characterization of the EPL reaction. (B) Denaturing SDS-PAGE analysis of the reverse Ni-IMAC purified NbHE4-NbEGFR-R_9_ bispecific endobody.

**Supplementary Figure 8.** Preparation of 2×NbEGFR-R_9_-PTD1 (EER_9_P1) endobody and 2×NbEGFR as the negative control. (A) Denaturing SDS-PAGE characterization of EPL reaction and purified EER_9_P1 endobody. (B) Denaturing SDS-PAGE characterization of EPL reaction and purified 2×NbEGFR-R_9_ bivalent endobody. (C) Denaturing SDS-PAGE characterization of EPL reaction and purified 2×NbEGFR bivalent nanobody.

**Supplementary Figure 9.** Nystatin nor EIPA does not inhibit the degradation of EGFR by EER_9_P1. (A-B) WB analysis revealed no inhibition of degradation by Nystatin (20 μM, A) nor EIPA (30 μM, B), suggesting caveolae and micropinocytosis independent ELS pathway for EER_9_P1 induced protein degradation (n = 3 experiments). One-sided independent Student’s *t*-test were used in all statistical analysis; ns: non-significant.

**Supplementary Figure 10.** Preparation of other PTD1-enhanced endobodies. (A-B) Preparation of 2×NbPD-L1-R_9_-PTD1 endobody and 2×NbPD-L1 bivalent nanobody as the negative control. Denaturing SDS-PAGE characterization of the EPL reactions (left) and reverse Ni-IMAC purified products (right). (C-D) Preparation of 2×NbHER2-R_9_-PTD1 endobody and 2×NbHER2 bivalent nanobody as the negative control. Denaturing SDS-PAGE characterization of the EPL reactions (left) and reverse Ni-IMAC purified products (right). (E-F) Preparation of 2×NbHE4-R_9_-PTD1 endobody and 2×NbHE4 bivalent nanobody as the negative control. Denaturing SDS-PAGE characterization of the EPL reactions (left) and reverse Ni-IMAC purified products (right).

**Supplementary Figure 11.** Other enhanced endobodies all demonstrates increased degradation potency. (A) 2×NbPD-L1-R_9_-PTD1 endobody triggers more potent degradation of PD-L1 in MDA-MB-231 cells. (B) The enhanced endobody 2×NbPD-L1-R_9_-PTD1 demonstrates higher degradation potency than other control constructs, as well as the blank control (PBS) and the standard NbPD-L1-R_9_ endobody (n = 3 experiments). (C) WB analysis revealed 2×NbHER2-R_9_-PTD1 to induce HER2 degradation at low-nanobody concentration in SK-BR-3 cells. (D) The enhanced endobody 2×NbHER2-R_9_-PTD1 demonstrates higher degradation potency than other control constructs, as well as the blank control (PBS) and the standard NbHER2-R_9_ endobody (n = 3 experiments). (E) WB analysis revealed that 2×NbHE4-R_9_-PTD1 induces HE4 degradation at low-nanobody concentration in OVCAR3 cells. (F) The enhanced endobody 2×NbHE4-R_9_-PTD1 demonstrates higher degradation potency than other control constructs, as well as the blank control (PBS) and the standard NbHE4-R_9_ endobody (n = 3 experiments). One-sided independent Student’s *t*-test were used in all statistical analysis; ns: non-significant.

**Supplementary Figure 12.** EER_9_P1 endobody suppresses growth of lung cancer xenograft tumor in vivo via intravenous administration. (A) Schematic overview of xenograft lung cancer mice model generation and drug administration in BALB/c nude mice. ~6×10^6^ A549 cells in 0.1 ml of PBS/Matrigel solution were subcutaneously injected into the axillary region of the BALB/c nude mice to allow tumor generation. Drugs were intravenously injected every two days. (B) Tumor growth curve of each individual mouse for PBS (blank), EER_9_P1 (3 mg∙kg^−1^), and EER_9_P1 (5 mg∙kg^−1^) injected groups (n = 3 mice per group). (C) Statistical quantification of the tumor growth for different groups. Statistical analysis: mean ± SD; Student’s *t*-test used; ns: non-significant. (D) Body weight curves for each group of mice. Mean ± SD shown. (E) Photographs of the dissected tumor of each mouse 15 days after the first drug injection. (F) Statistical analysis of the tumor weight of each mice group: Mean ± SD; Student’s *t*-test used. (G) Representative WB analysis result for assessing the stability of EER_9_P1 (100 μg∙mL^−1^) in serum at 37 °C over time.
